# Reply to Letter by Tellier et al., ‘Scientific refutation of ESHG statement on embryo selection’

**DOI:** 10.1038/s41431-022-01241-4

**Published:** 2022-12-01

**Authors:** Francesca Forzano, Olga Antonova, Angus Clarke, Guido de Wert, Sabine Hentze, Yalda Jamshidi, Yves Moreau, Markus Perola, Inga Prokopenko, Andrew Read, Alexandre Reymond, Vigdis Stefansdottir, Carla van El, Maurizio Genuardi, Executive Committee of the European Society of Human Genetics, Maurizio Genuardi, Borut Peterlin, Carla Oliveira, Karin Writzl, Gunnar Douzgos Houge, Public and Professional Policy Committee of the European Society of Human Genetics, Christophe Cordier, Heidi Howard, Milan Macek, Béla Melegh, Alvaro Mendes, Dragica Radojkovic, Emmanuelle Rial-Sebbag, Fiona Ulph

**Affiliations:** 1 Clinical Genetics Department, Guy’s and St Thomas NHS Foundation Trust, London, UK; 2 Department of Medical Genetics, Medical University of Sofia, Sofia, Bulgaria; 3 Division of Cancer and Genetics, School of Medicine, Cardiff University, Cardiff, Wales UK; 4 Maastricht University, Maastricht, The Netherlands; 5 Practice for Human Genetics, Heidelberg, Germany; 6 Genetics Research Centre, Molecular and Clinical Sciences Institute, St George’s University of London, London, UK; 7 University of Leuven ESAT-STADIUS, B-3001 Leuven, Belgium; 8 Finnish Institute for Health and Welfare (THL), Biomedicum 1, Haartmaninkatu 8, 00290 Helsinki, Finland; 9 Department of Clinical & Experimental Medicine, University of Surrey, Guildford, UK; 10 UMR 8199 – EGID, Institut Pasteur de Lille, CNRS, University of Lille, F-59000 Lille, France; 11 People-Centred Artificial Intelligence Institute, University of Surrey, Guildford, UK; 12 Centre for Genomic Medicine, St Mary’s Hospital, M13 0JH Manchester, England; 13 Center for Integrative Genomics, University of Lausanne, CH- 1015 Lausanne, Switzerland; 14 Department of Genetics and Molecular Medicine, Landspitali University Hospital, Reykjavik, Iceland; 15 Section Community Genetics, Department of Clinical Genetics and Amsterdam Public Health Research Institute, Amsterdam UMC, Vrije Universiteit Amsterdam, Amsterdam, The Netherlands; 16 UOC Genetica Medica, Dipartimento di Scienze di Laboratorio e Infettivologiche, Fondazione Policlinico Universitario A. Gemelli IRCCS, Rome, Italy; 17 Sezione di Medicina Genomica, Dipartimento di Scienze della Vita e Sanità Pubblica, Università Cattolica del Sacro Cuore, Rome, Italy; 18 Clinical Institute for Genomic Medicine, University Medical Center Ljubljana, Ljubljana, Slovenia; 19 Institute of Molecular Pathology and Immunology of the University of Porto (Ipatimup), University of Porto, PT Porto, Portugal; 20 Department of Pathology, Faculty of Medicine, University of Porto, PT Porto, Portugal; 21 Instituto de Investigação e Inovação em Saúde (i3S), University of Porto, PT Porto, Portugal; 22 Department of Medical Genetics, Haukeland University Hospital, 5021 Bergen, Norway; 23 Department of Genetics, SYNLAB Suisse SA, Chemin d’Entre Bois 21, 1018 Lausanne, Switzerland; 24 Medical Ethics, Lund University, Uppsala, Sweden; 25 Chalmers University (part of GENIE initiative), Uppsala, Sweden; 26 Department of Biology and Medical Genetics, 2nd Faculty of Medicine, Charles University and Motol University Hospital, V Uvalu 84, Prague, CZ15006 Czech Republic; 27 Department of Medical Genetics, University of Pécs, Szigeti 12., H-7624 Pécs, Hungary; 28 UnIGENe and Centre for Predictive and Preventive Genetics, IBMC—Institute for Molecular and Cell Biology, i3S—Instituto de Investigação e Inovação em Saúde, Universidade do Porto, Porto, Portugal; 29 Laboratory for Molecular Biology, Institute of Molecular Genetics and Genetic Engineering, University of Belgrade, Belgrade, Serbia; 30 CERPOP, UMR 1295, Inserm, Université de Toulouse-Université Paul Sabatier-Toulouse III, Responsable Equipe BIOETHICS: Trajectoires d’innovations en santé:enjeux bioéthiques et sociétaux, Toulouse, France; 31 Plateforme Sociétale “Génétique et Société, GIS Genotoul, Génopole Toulouse Midi-Pyrénées, 37, allées Jules Guesde, 31073 Toulouse Cedex, France; 32 Manchester Centre of Health Psychology, Division of Psychology and Mental Health, School of Health Sciences, Manchester Academic Health Science Centre, University of Manchester, Coupland Street, Manchester, M13 9PL UK

## To the Editor:

We would like to thank the authors for their letter addressing our recent policy paper on PGT-P, as this provides us with an additional opportunity to clarify our position.

Tellier et al. criticise the selection of papers we have cited, considering them not sufficiently representative of the wealth of literature on this subject, so that, according to them, we have not correctly represented the ‘scientific consensus’ and ‘potential utility’ of the technology.

It is important to emphasise that our paper does not aim to address the research underlying polygenic risk scores (PRSs) in general, nor the full range of potential screening and clinical applications, but only those PRSs applied to embryo selection and ranking (so-called PGT-P). We would like to reassure Tellier et al. that we have considered a much larger body of literature than just the papers we have referred to. As one might expect, we selected the papers that are the most relevant and important for the very specific scope of our policy paper.

We are quite puzzled, however, by the view expressed by the authors of the letter about a ‘scientific consensus’ regarding the clinical application of PRSs to embryo selection. Indeed, if a consensus can be said to exist, it seems to us to be very much contrary to the views of Tellier et al. In 2021 and 2022, the European Society of Human Genetics [1], the American College of Medical Genetics [2], the European Society of Human Reproduction and Embryology [3], the International Society of Psychiatric Genetics [4] and the Polygenic Risk Score Task Force of the International Common Disease Alliance [5] all released statements concordant in their opinion that preimplantation or prenatal testing for common disorders using PRSs is not yet appropriate for clinical use.

While we agree with the authors that PGT-P might be able to identify some ‘risk outliers’ among sibling IVF embryos, we disagree with their claim that the differences among sibling IVF embryos will be, on average, significant enough to enable meaningful, clinically useful selection or ranking. The lack of any likely substantial net effect on traits such as duration of education is indeed one of the key points made by Turley et al. [6]. The latter is cited by Tellier et al. as if it supports their own views, but we read it very differently from them. Even the paper they cite by Lello et al. (whose authorship overlaps with the letter), while demonstrating some ability to distinguish PRSs of siblings, fails to produce convincing evidence that this would be of any clinical utility in testing embryos [7]. Nor would there be any path to determine the accuracy of any ‘predictions’ made on the basis of such claims. Furthermore, it is quite strange that yet another paper cited in their letter [8] concludes that ‘screening human embryos for polygenic traits has limited utility’. Tellier et al. are maybe striving to cite the literature fairly, even if it undermines their position. Of course, if selection based on PRSs were to be applied for more than one trait at the same time, any reason to believe it could be employed in a useful way becomes even more remote in most family-specific circumstances.

Another point where we disagree with the authors is their statement that the selection they can achieve would confer a disease risk reduction comparable to that of embryo selection for monogenic disease. We disagree with this for two reasons. First, such large effects of PRSs are not usually available within a single nuclear family [6], nor does the paper by Lello et al. support this [7]. Second, what would be at stake is a relative increase or reduction of risk compared with the general population for a common disorder, though it will never be possible to exclude the development of that condition in the chosen embryo. Conflating the calculation of risks for common multifactorial disorders with that for rare monogenic disorders, even where they have a reduced penetrance, is both mistaken and misleading.

The authors use as a supportive argument for the use of PGT-P the fact that ‘roughly 50% of US IVF embryos undergo some form of genetic screening today’. We hope that the authors would concur that performing one form of screening does not automatically entail endorsing the use of a second, particularly if it has not been adequately assessed. Though aneuploidy screening in preimplantation embryos (PGT-A) has been introduced in many (private) clinics, this screening is not without its critics. In fact, a relatively recent Cochrane review [9] has concluded that the currently available evidence is insufficient to support PGT-A in routine clinical practice. This apparent conundrum highlights yet further our still limited knowledge of embryo physiology and development, and the differences in testing an early embryo as compared to a foetus or a newborn.

We are glad to know that the authors would welcome an open scientific discussion on the merits of PGT-P, and we would hope this would, at the same time, include addressing the relevant ethical issues, such as ramping up false expectations as to what can be achieved through the application of unevaluated new technologies, which might lead to ill-advised management of the couple’s reproductive journey and potentially to financial exploitation. We strongly support this call for a frank debate, with the caveat that this should precede, and not follow, the introduction of this test in the clinic.

## References

[1] Forzano F, Antonova O, Clarke A, de Wert G, Hentze S, Jamshidi Y (2022). The use of polygenic risk scores in pre-implantation genetic testing: an unproven, unethical practice. Eur J Hum Genet.

[2] ACMG Board of Directors Direct-to-consumer prenatal testing for multigenic or polygenic disorders: a position statement of the American College of Medical Genetics and Genomics (ACMG). Genet Med. 2021;23:2027–8. 10.1038/s41436-021-01247-1 34183790

[3] ESHRE. ESHRE supports the position of ESHG on embryo selection based on polygenic risk scores. 2022. https://www.eshre.eu/Europe/Position-statements/PRS.

[4] ISPG Board Advisory on the use of Polygenic Risk Scores to screen embryos for adult mental health conditions. 2021. https://ispg.net/ethics-statement/.

[5] Polygenic Risk Score Task Force of the International Common Disease Alliance. (2021). Responsible use of polygenic risk scores in the clinic: potential benefits, risks and gaps. Nat Med.

[6] Turley P, Meyer MN, Wang N, Cesarini D, Hammonds E, Martin AR (2021). Problems with using polygenic scores to select embryos. N Eng J Med.

[7] Lello L, Raben TG, Hsu SDH (2020). Sibling validation of polygenic risk scores and complex trait prediction. Sci Rep.

[8] Karavani E, Zuk O, Zeevi D, Barzilai N, Stefanis NC, Hatzimanolis A (2019). Screening human embryos for polygenic traits has limited utility. Cell.

[9] Cornelisse S, Zagers M, Kostova E, Fleischer K, van Wely M, Mastenbroek S. Preimplantation genetic testing for aneuploidies (abnormal number of chromosomes) in in vitro fertilisation. Cochrane Database Syst Rev. 2020;9:CD005291. 10.1002/14651858.CD005291.pub3 PMC8094272 32898291

