# Correction to: Reply to Letter by Tellier et al., ‘Scientific refutation of ESHG statement on embryo selection’

**DOI:** 10.1038/s41431-022-01263-y

**Published:** 2022-12-19

**Authors:** Francesca Forzano, Olga Antonova, Angus Clarke, Guido de Wert, Sabine Hentze, Yalda Jamshidi, Yves Moreau, Markus Perola, Inga Prokopenko, Andrew Read, Alexandre Reymond, Vigdis Stefansdottir, Carla van El, Maurizio Genuardi, Executive Committee of the European Society of Human Genetics, Maurizio Genuardi, Borut Peterlin, Carla Oliveira, Karin Writzl, Gunnar Douzgos Houge, Public and Professional Policy Committee of the European Society of Human Genetics, Christophe Cordier, Heidi Howard, Milan Macek, Béla Melegh, Alvaro Mendes, Dragica Radojkovic, Emmanuelle Rial-Sebbag, Fiona Ulph

**Affiliations:** 1 Clinical Genetics Department, Guy’s and St Thomas NHS Foundation Trust, London, UK; 2 Department of Medical Genetics, Medical University of Sofia, Sofia, Bulgaria; 3 Division of Cancer and Genetics, School of Medicine, Cardiff University, Cardiff, Wales UK; 4 Maastricht University, Maastricht, The Netherlands; 5 Practice for Human Genetics, Heidelberg, Germany; 6 Genetics Research Centre, Molecular and Clinical Sciences Institute, St George’s University of London, London, UK; 7 University of Leuven ESAT-STADIUS, B-3001 Leuven, Belgium; 8 Finnish Institute for Health and Welfare (THL), Biomedicum 1, Haartmaninkatu 8, 00290 Helsinki, Finland; 9 Department of Clinical & Experimental Medicine, University of Surrey, Guildford, UK; 10 UMR 8199 – EGID, Institut Pasteur de Lille, CNRS, University of Lille, F-59000 Lille, France; 11 People-Centred Artificial Intelligence Institute, University of Surrey, Guildford, UK; 12 Centre for Genomic Medicine, St Mary’s Hospital, M13 0JH Manchester, England; 13 Center for Integrative Genomics, University of Lausanne, CH- 1015 Lausanne, Switzerland; 14 Department of Genetics and Molecular Medicine, Landspitali University Hospital, Reykjavik, Iceland; 15 Section Community Genetics, Department of Clinical Genetics and Amsterdam Public Health Research Institute, Amsterdam UMC, Vrije Universiteit Amsterdam, Amsterdam, The Netherlands; 16 UOC Genetica Medica, Dipartimento di Scienze di Laboratorio e Infettivologiche, Fondazione Policlinico Universitario A. Gemelli IRCCS, Rome, Italy; 17 Sezione di Medicina Genomica, Dipartimento di Scienze della Vita e Sanità Pubblica, Università Cattolica del Sacro Cuore, Rome, Italy; 18 Clinical Institute for Genomic Medicine, University Medical Center Ljubljana, Ljubljana, Slovenia; 19 Institute of Molecular Pathology and Immunology of the University of Porto (Ipatimup), University of Porto, PT Porto, Portugal; 20 Department of Pathology, Faculty of Medicine, University of Porto, PT Porto, Portugal; 21 Instituto de Investigação e Inovação em Saúde (i3S), University of Porto, PT Porto, Portugal; 22 Department of Medical Genetics, Haukeland University Hospital, 5021 Bergen, Norway; 23 Department of Genetics, SYNLAB Suisse SA, Chemin d’Entre Bois 21, 1018 Lausanne, Switzerland; 24 Medical Ethics, Lund University, Uppsala, Sweden; 25 Chalmers University (part of GENIE initiative), Uppsala, Sweden; 26 Department of Biology and Medical Genetics, 2nd Faculty of Medicine, Charles University and Motol University Hospital, V Uvalu 84, Prague, CZ15006 Czech Republic; 27 Department of Medical Genetics, University of Pécs, Szigeti 12., H-7624 Pécs, Hungary; 28 UnIGENe and Centre for Predictive and Preventive Genetics, IBMC—Institute for Molecular and Cell Biology, i3S—Instituto de Investigação e Inovação em Saúde, Universidade do Porto, Porto, Portugal; 29 Laboratory for Molecular Biology, Institute of Molecular Genetics and Genetic Engineering, University of Belgrade, Belgrade, Serbia; 30 CERPOP, UMR 1295, Inserm, Université de Toulouse-Université Paul Sabatier-Toulouse III, Responsable Equipe BIOETHICS: Trajectoires d’innovations en santé:enjeux bioéthiques et sociétaux, Toulouse, France; 31 Plateforme Sociétale “Génétique et Société, GIS Genotoul, Génopole Toulouse Midi-Pyrénées, 37, allées Jules Guesde, 31073 Toulouse Cedex, France; 32 Manchester Centre of Health Psychology, Division of Psychology and Mental Health, School of Health Sciences, Manchester Academic Health Science Centre, University of Manchester, Coupland Street, Manchester, M13 9PL UK

Correction to: *European Journal of Human Genetics* 10.1038/s41431-022-01241-4, published online 01 December 2022

In this article the affiliation details for Carla Oliveira should have been as follows.

Carla Oliveira^19, 20, 21^

^19^Institute of Molecular Pathology and Immunology of the University of Porto (Ipatimup), University of Porto, PT, Porto, Portugal

^20^Department of Pathology, Faculty of Medicine, University of Porto, PT, Porto, Portugal

^21^Instituto de Investigação e Inovação em Saúde (i3S), University of Porto, PT, Porto, Portugal

Due to that, affiliations numbers for other authors were renumbered accordingly.

The original article has been corrected.

